# Pneumo-PET-CT: Initial Results of This Novel Technique on the Evaluation of Esophageal and Gastric Tumors with Anatomic-Surgical Correlation

**DOI:** 10.1155/2019/4123851

**Published:** 2019-02-05

**Authors:** Facundo N. Diaz, Marina Ulla, Jose M. Lastiri, Fernando G. Wright, Demetrio Cavadas

**Affiliations:** ^1^Diagnóstico por Imágenes, Hospital Italiano de Buenos Aires, Juan Domingo Peron 4190 (C1199AAB), Ciudad Autónoma de Buenos Aires, Argentina; ^2^Universidad de Buenos Aires, Facultad de Medicina, II Cátedra de Anatomía, Buenos Aires, Argentina

## Abstract

We present the initial results of a novel hybrid scanning-based technique that combines pneumo-computed tomography (PNCT) with positron emission tomography (PET) using 2-(fluorine-18) fluoro-2-deoxy-D-glucose (FDG). We denominate it pneumo-PET-CT. The focus of our discussion will be on the description of the pneumo-PET-CT technique and the interpretation criteria with emphasis on its benefits and applications in the presurgical and postneoadjuvant therapy evaluation of esophageal, esophagogastric junction (EGJ), and gastric tumors.

## 1. Introduction

Esophageal cancer is the eighth most frequent cancer worldwide, and adenocarcinoma is currently the most common in Western countries, with a significant increase in its incidence in recent years in relation to gastroesophageal reflux disease and obesity. Other tumor types, such as lymphoma, spindle cell carcinoma, neuroendocrine tumors, and gastrointestinal stromal tumors (GIST), are very uncommon [1].

Esophageal adenocarcinoma presents an aggressive behavior and is usually diagnosed in advanced stages. Despite recent advances in diagnosis and treatment, the prognosis remains poor and represents the sixth leading cause of cancer death in the world, with a 5-year survival rate of less than 20% [1, 2].

Also, gastric cancer is the fifth most common malignancy in the world, and a substantial portion of patients with newly diagnosed gastric cancer has distant metastases (M1 disease), which incurs a very poor prognosis [3].

For these reasons, early diagnosis and accurate staging of esophageal cancer are both essential for therapeutic strategy planning [2]. While surgery is the mainstay of treatment of this disease, the utilization of chemoradiation, either used as postsurgical treatment or as a neoadjuvant therapy, has become a standard practice in the United States [3–5].

The TNM staging system for cancer in the esophagus and EGJ has been revised in the eighth edition, published in 2017 by the American Joint Committee of Cancer (AJCC), and is unified with the staging of stomach cancer, also published in the same manual [6].

Despite the importance of pretreatment staging, no single test or combination of tests for staging esophageal cancer has been accepted as the standard of care [8, 9]. Each one has its strengths and weaknesses.

Conventional endoscopy (CE) with biopsy is the established primary diagnostic method. Its strengths are that it is a simple diagnostic and available method, it has a low cost, and it is of rapid evaluation. Its weaknesses are that it is an invasive method and inaccurate for staging or characterizing the lesion. High-grade stenoses are difficult to be overcome by endoscopy [8, 9].

Barium studies (BS) are conventionally used; they share some advantages of CE adding an evaluation of the long axis of the esophagus. However, BS are not a diagnostic method and also are inaccurate for staging [7–9].

Endoscopic ultrasound (EUS) has been found to be the most accurate imaging modality for local T staging and locoregional lymphadenopathy, with a T staging accuracy of 75%-85% and N stage accuracy of 65%-75% [2]. However, EUS is unable to detect distant metastases, which is the most crucial factor in determining when cancer will be resectable at surgery, and it provides no information for surgical planning.

Another weakness of the method is the inability of current probes to cross-stenotic tumors.

Positron emission tomography (PET) scan has been more recently used in staging esophageal and gastric cancer [6]. Examining the biologic function by examining the uptake of glucose, PET has the strength to evaluate both the locoregional and distant spread of tumor. PET weaknesses are its low availability and also lack of information for surgical planning [8]. Conventional CT is a simple, available, and rapid evaluation method. It has been the first staging method used for staging esophageal and gastric cancer, with high accuracy for the detection of liver metastases but reduced ability to accurately detect T4 disease because of the local invasion or local lymph node spread [8]. Conventional CT weakness for hollow organ assessment is that in the absence of lumen distension, the organ wall may be collapsed.

Given this context and with the help of a new equipment, we propose to fuse a distension-based CT technique called pneumo-CT (PNCT) and PET-CT scan with FDG.

The purpose of this article is to describe this pneumo-PET-CT technique and the interpretation criteria that we use according to the eighth edition of the AJCC staging manual and to illustrate it with some cases with anatomic-surgical correlation.

## 2. Esophageal and Gastric Distension

PNCT associated with virtual CT endoscopy was previously described by one of the authors as the pneumo-64-MDCT technique [2, 7–9]. With this technique, a maximum lumen distension is achieved. Intraluminal and intramural pathology, as well as the shape, anatomic location, and size of intraluminal masses, or esophageal wall thickening is better showed [2].

The PNCT method showed usefulness for staging and therapeutic strategy. For esophageal tumors which are considered unresectable, the definition of both the upper and lower limits of the tumor in the longitudinal axis allows the surgeon to determine the length of the stent graft or the need for a valved stent [2].

Stomach distension led to an adequate definition of both the upper and lower borders of the lesion in tumors located in the GE junction, which in turn was useful to plan the surgical approach, where the depiction of the tumor's anatomic location determines the surgical strategy [2].

Given these reasons, we present a one shot study technique (from now on referred to as Pneumo-PET-CT) for those patients with clinical indication of performing both CT and PET CT with the PNCT distension technique. Its potential usefulness for presurgical and postneoadjuvant therapy evaluation of esophageal, EGJ, and gastric tumors will be showed with an anatomopathological evaluation.

## 3. Materials and Methods

### 3.1. Patients

This is a descriptive study with a retrospective data analysis, approved by the ethical committee of our institution.

Between July and May 2018, 44 adult patients (31 males, 13 females), mean age 62.02 years (range 39-90) with suspected or confirmed diagnosis of esophageal (including EGJ) or malignant gastric tumors, were studied with the pneumo-PET-CT technique. All patients were over 18 years old and signed an informed consent for this study.

A total of 6 patients were excluded from the final analysis due to the absence of histological confirmation of the lesion (from biopsy or surgical pathology).

### 3.2. Technique

Patients with an 8-hour preprocedural fasting are received by a nurse, a peripheral venous line is placed, and blood glucose level is measured. A level of less than 150 mg/dl is desirable.

A typical dose of 10 mCi of FDG is injected intravenously, and image acquisition is initiated approximately 60 minutes after.

They all were informed about the procedure and signed the corresponding informed consent.

Just before being positioned on the PET-CT table, a 16-French Foley catheter is placed right below the cricopharyngeal muscle under local gel anesthesia. Given the extended time of PET scan, we prefer the transnasal introduction over the transoral if there are no contraindications (i.e., recent septum surgery, recent epistaxis, or craniofacial malformations).

To achieve optimal esophageal, GEJ, and gastric distension, we use

- An intravenous antispasmodic (hyoscine N-butylbromide, 20 mg) administered to avoid esophageal spasm that may simulate wall thickening or stenosis
- A continuous CO_2_ supplied and sustained during the acquisition with a pressure between 12 and 15 mmHg by a CO_2_ pump (Protocol pump, PROTOCO2L, E-Z-EM Inc.)
- A warning to the patients that slight discomfort may be experienced due to the esophageal distension and instruction to hold the air and avoid burping during the procedure

### 3.3. CT and PET Protocols

All studies are performed at our institution with a PET Biograph 20 mCT Excel (Siemens AG). Anterior scout view is obtained to program both tomographic and PET acquisitions.

A two-phase CT is performed, one acquisition is nonenhanced with inspiratory apnea from the base skull to the iliac crest. It helps to evaluate the correct distension of the esophagus, EGJ, and the stomach and to the proper visualization of the lungs. The other contrast-enhanced acquisition is performed with the patient breathing quietly from the top of the skull to the thighs following the injection of a nonionic iodinated contrast (Preray, iopamidol 370 mg/ml) at a dose of 1 ml/kg is infused using an automatic injection pump at a flow of 2.5 ml if there are no contraindications. No oral contrast is used.

CT acquisitions are performed with 1.0 mm slice thickness, 1.0 mm reconstruction interval, pitch of 0.8, 200 mAs/slice, 0.7 rotation time, 120 kV, and a 512 × 512 matrix. The acquisition time is approximately 20 seconds, and the typical effective radiation dose is 35-40 mSv.

PET is performed following the CT acquisitions using cerium-doped lutetium oxyorthosilicate (LSO) crystals from the top of the skull to the thighs with the patient breathing quietly. Usually, a set of 10 bed positions is planned with a 50% overlapping between them and a 2-minute duration for each bed position.

The total time required to complete the whole study (CT and PET) is approximately 25 minutes.

The obtained isotropic images are sent to both syngo.via workstation and Vitrea advanced working station for PET-CT fusion and postprocessing technique.

As the first step of postprocessing of images, multiplanar reconstructions (MPR) and curved MPR are performed with different window settings to characterize the primary lesion. A description of the shape, location, size, and thickness of the lesion is done.

To allow a better visualization of the primary lesion, we generate 3D reconstructions with different window settings (surface-shaded and transparent mode similar to the images obtained in single and double-contrast barium studies).

At last, we can virtually introduce inside the esophagus lumen and generate endoluminal views that show the lesion morphology, generating virtual endoscopy images.

## 4. Results and Discussion

From 44 studies, a total of 6 were excluded from the final analysis due to the absence of histological confirmation of the lesion (from biopsy or surgical pathology). Two of them located in the middle third of the esophagus were squamous cell carcinoma. From 8 cases of tumors located between 1 and 5 cm above the EGJ (Siewert type 1 topography), 8 were adenocarcinoma. From 8 cases located from 1 cm above to 2 cm below the EGJ (Siewert type 2 topography), 6 were adenocarcinoma, 1 was squamous cell carcinoma, and 1 was a neuroendocrine tumor. The 3 cases located between 2 and 5 cm below the EGJ (Siewert type 3 topography) were adenocarcinoma, and finally, from 17 cases located at the stomach (distal to type 3 Siewert topography), 14 were adenocarcinoma, 1 was metastatic disease from breast carcinoma, 1 was lymphoma, and finally, 1 was a spindle cell carcinoma.

### 4.1. Image Analysis and Evaluation Criteria

Images are analyzed and processed by a multidisciplinary team (a radiologist and a nuclear medicine specialist with a surgeon and an oncologist). It allows a better interpretation and avoids eventually reporting differences that may happen if they work separately.

For establishing an accurate image analysis according to the eighth edition of TNM from AJCC/UICC for both esophagus and gastric tumors, we follow the next criteria.

### 4.2. Esophagus and Esophagogastric Junction

**Figure 1:**
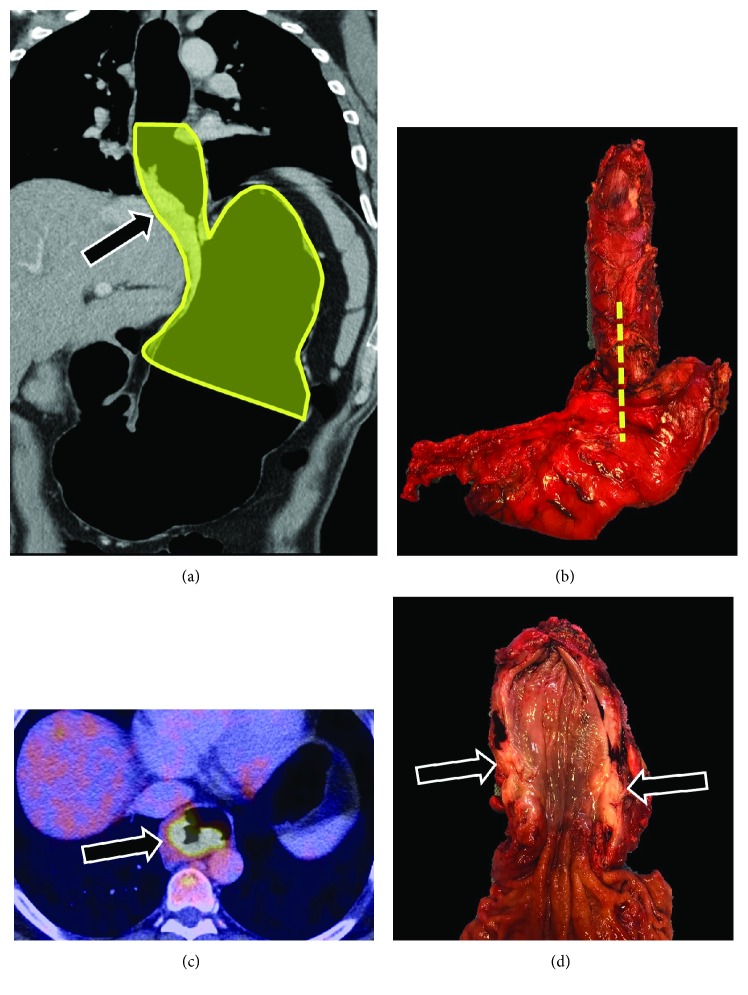
A 68-year-old male patient with EGJ confirmed adenocarcinoma (black arrows) showing a coronal curved reconstruction (a) with the planned surgery (yellow highlight), the axial pneumo-PET-CT fusion showing hypermetabolism of the tumor, and the surgical piece (b, closed, and d, opened).

Tumors in the EGJ are staged as esophageal cancer if the tumor epicenter is within the lower thoracic esophagus, or involving the EGJ, and have their epicenter within the proximal 2 cm of the cardia (Siewert type I/II) (Figure 1). Cancers whose epicenter is more than 2 cm distal from the EGJ, even if the EGJ is involved, will be staged using the stomach cancer TNM [6].

These EGJ tumors can also be divided into three groups according to Siewert's modified classification (for adenocarcinoma only): type 1 if the tumor's epicenter is between 1 and 5 cm above the EGJ, type 2 with the epicenter 1 cm above to 2 cm below the EGJ, and type 3 with the epicenter between 2 and 5 cm below the EGJ [1].

The degree of primary tumor invasion is represented by the T classification, which provides details regarding local tumor invasion into the esophageal wall and advanced invasion into adjacent structures [6].

A thickness greater than 5 mm with a distended esophagus is considered abnormal [6] and could be T1 or T2.

Adventitial penetration may appear as a defined abnormal soft tissue around the tumor [6]. We considered it T3 if the fat planes with adjacent structures are preserved.

T4a tumors invade adjacent structures such as the pleura, peritoneum, pericardium, or diaphragm, and they are resectable. On the other hand, T4b tumor invades the aorta, carotid vessels, azygos vein, trachea left main bronchus, or vertebral body and are unresectable cancers [6].

Tumoral infiltration is suspected if there is alteration of the fat plane with those structures, and if the contact with the aorta is more than 1/4 of the aortic circumference, or if there is obliteration of the fat triangle between the esophagus, aorta, and adjacent vertebral body aortic's tumoral invasion is suspected [1, 6].

The N classification, which considers regional lymph node involvement, is the most important prognostic factor in esophageal cancer, is based on regional nodes (from the paraesophageal cervical nodes to the celiac nodes), and is determined by the absence (N0) or presence of one or two cancer-positive nodes (N1), three to six (N2), or seven or more (N3) [6].

We considered a pathological lymph node when it is larger than 10 mm for abdominal nodes, or larger than 5 mm for supraclavicular nodes [6]. Loosening of the normal morphology (smooth and well-defined border, uniform homogeneous attenuation, and a central fatty hilum), central necrosis, and marked or heterogeneous enhancement should be considered pathological too. With PET-FDG, a normal-size node can show hypercaptation of FDG and may be considered pathological.

M classification is designated M0 or M1 according to the absence or presence of distant metastasis, respectively [6].

M classification is an important factor in determining operability, and metastases are most commonly diagnosed in the liver (35%), followed by the lungs (20%), bones (9%), adrenal glands (5%), and, rarely, peritoneum and the brain (1, 6). PET-FDG may show distant metastases difficult to see on conventional CT.

Those findings are summarized in Table 1.

### 4.3. Stomach

**Figure 2:**
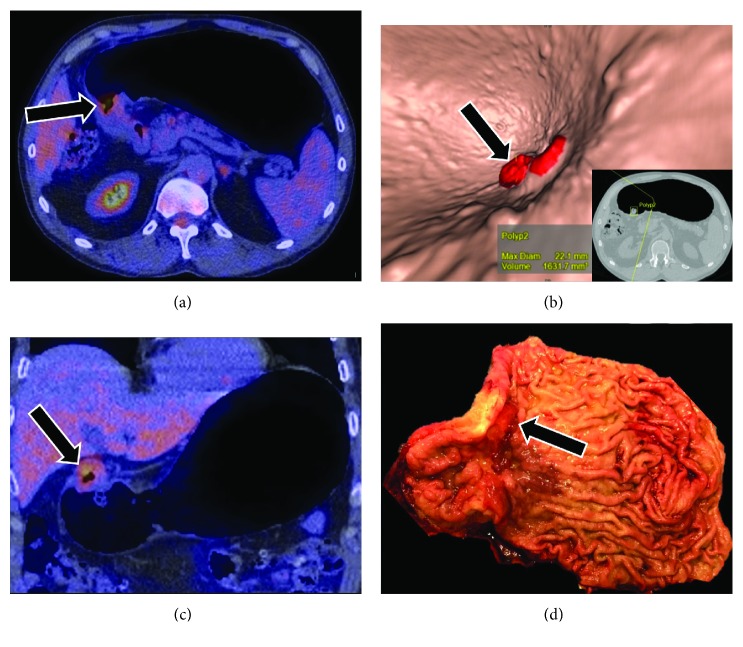
A 54-year-old female patient with gastric adenocarcinoma (black arrows) showing an axial pneumo-PET-CT fusion with hypermetabolism (a) and endoscopic reconstruction (b) and the correlation between coronal pneumo-PET-CT fusion (c) and the surgically opened piece (d).

**Figure 3:**
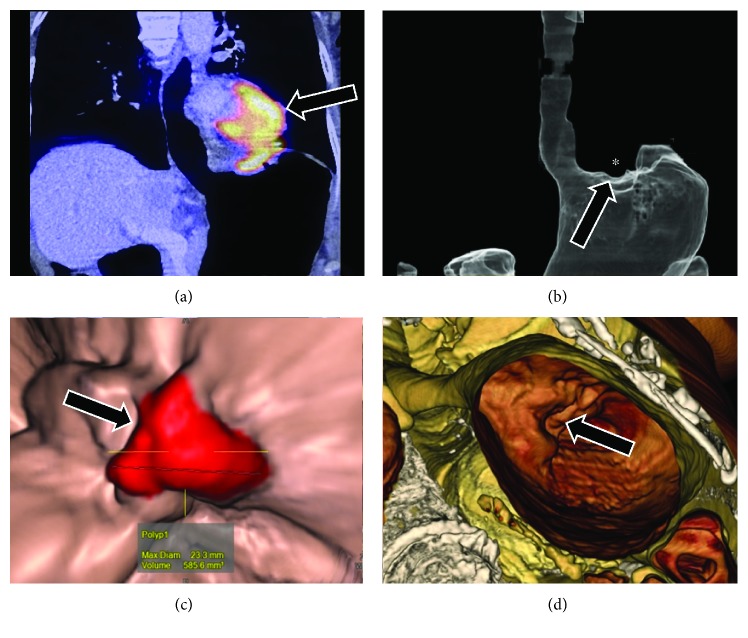
A 79-year-old male patient with gastric adenocarcinoma seen as a fundic mass (black arrows) with heterogeneous hypermetabolism (a) and virtual reconstruction showing the air fill (b) and endoscopic views (c and d).

In T1 and T2 lesions, invasion is limited to the gastric wall, whose outer border may be smooth (Figure 2); in T3 lesions, the serosal contour becomes blurred and strand-like areas of increased attenuation may be seen extending into the perigastric fat (Figure 3); and in T4 lesions, tumor spread frequently occurs via ligamentous and peritoneal reflections to adjacent organs [4]. T4a tumor only invades the visceral peritoneum, and T4b invades adjacent structures [7].

A gastric mass that abuts an adjacent organ and absence of the fat plane between the mass and the organ are suggestive of but not diagnostic for organ invasion [4].

PET information is not helpful in T staging because it is a functional imaging modality. In primary tumor detection, variable levels of FDG uptake have been found. Gastric adenocarcinomas, such as mucinous carcinoma, signet ring cell carcinoma, and poorly differentiated adenocarcinomas, tend to show significantly lower FDG uptake than do other histologic types of gastric cancer [4.]

PET-FDG has greater sensitivity than CT in the evaluation of peritoneal carcinomatosis. Two patterns of FDG uptake are known to be indicators of peritoneal metastasis:

- Diffuse uptake spreading uniformly throughout the abdomen and pelvis, obscuring visceral outlines (normal serpiginous pattern of the large and small bowel and physiologic hepatic and splenic uptake)
- Discrete foci of uptake located randomly and anteriorly within the abdomen or dependently within the pelvis and unrelated to solid viscera or nodal stations [4]

As for esophageal cancer, CT-positive nodes are identified by the size, shape, and enhancement pattern (i.e., more than 8–10 mm along the short axis, nearly round shape, central necrosis, and marked or heterogeneous enhancement).

N staging is based on the number of positive nodes: N1, metastasis in one or two regional lymph nodes; N2, metastasis in three to six nodes; and N3, metastasis in seven or more lymph nodes [7].

The regional lymph nodes of the stomach are classified into four compartments according to the Japanese Research Society for Gastric Cancer (JRSGC):

- Compartment I includes the perigastric lymph nodes (stations 1–6)
- Compartment II includes lymph nodes along the left gastric artery (station 7) and common hepatic artery (station 8), around the celiac axis (station 9), at the splenic hilum (station 10), and along the splenic artery (station 11)
- Compartment III includes lymph nodes in the hepatoduodenal ligament (station 12) at the posterior aspect of the head of the pancreas (station 13), and at the root of the mesentery (station 14). When the cancer is located in the lower third of the stomach, the lymph nodes along the splenic artery are classified as compartment III nodes
- Compartment IV includes lymph nodes along the middle colic vessels (station 15) and the paraaortic lymph nodes (station 16) [4]

Solid organ metastasis is uncommon in primary gastric cancers at the time of initial diagnosis, but its detection is important in treatment planning most commonly involving the liver because the portal vein drains the stomach. [3, 5].

Other less common sites of hematogenous spread include the lungs, adrenal glands, and skeleton, in the case of ovarian metastasis (Krukenberg tumor) [3].

PET-FDG is the most sensitive noninvasive imaging modality for the diagnosis of hepatic metastases from gastric and esophageal cancers. Theoretically, a small liver metastasis may be missed at CT but well seen at PET-FDG, and also extra abdominal lymph node metastasis can also be detected with PET [4].

Those findings are summarized in Table 2.

Until now, 20 of these patients went under surgery, and the preliminary results showed good correlation between pneumo-PET-CT radiological and pathological TNM staging, especially for differentiation between T2/3 and T4, and both N and M staging, with reduction of both radiation and iodinated-contrast media exposure.

We have not experienced adverse effects specific to the CO_2_ insufflation or iodinated contrast.

## 5. Conclusions

Compared to the single performance of both CT and PET-CT, this technique allows a simultaneous noninvasive assessment of the esophageal and gastric wall and distant metastatic disease (including the PET-FDG benefits) in a sole imaging method, minimizing both radiation and iodinated-contrast media exposure and reducing costs for the health system.

The combination of two- and three-dimensional and virtual endoscopic image analysis provides the advantage of being useful in therapeutic decision making and surgical planning.

As the main limitation, this preliminary report is based on a small group of patients, so further analysis with a more significant cohort of patients will be needed to validate this method and to determine the utility of this technique in the evaluation of chemotherapy and or radiotherapy.

In conclusion, pneumo-PET-CT emerges as a useful one-shot study technique in the clinical stratification and surgical planning of a broad spectrum of tumors within the esophagus, EGJ, and the stomach giving clinical information about advanced local invasion (T3/4), lymph node involvement (N), and distant spread (M).

## Figures and Tables

**Table 1:** Summary of radiological findings in a TNM approaching for esophageal cancer.

TNM approaching for esophageal cancer		PNCT findings	PET findings
T (primary tumor)	Is (in situ)	Carcinoma in situ. Could be normal at pneumo-PET-CT	
	1	Wall thickness (>5 mm)	Hypercaptation of FDG (it depends on the size of the tumor)
	2		
	3	Ill-defined abnormal tissue around the tumor (fat planes preserved)	
	4	Obliteration of fat planes with adjacent structures: 4a: the pleura, peritoneum, pericardium, or diaphragm 4b: the aorta, carotid vessels, azygos vein, trachea left main bronchus, or vertebral body	

N (lymph node involvement)	(0) Absence	Normal lymph nodes (smooth, well-defined border, uniform homogeneous attenuation, and a central fatty hilum, moderate and homogeneous enhancement, no FDG uptake)	
	(1) One or two nodes	Larger than 10 mm for abdominal nodes, or larger than 5 mm for supraclavicular nodes. Morphology alterations, central necrosis, or marked or heterogeneous enhancement should also be considered	Hypercaptation of FDG (even in normal-sized nodes should be considered pathological)
	(2) Three to six nodes		
	(3) Seven or more nodes		

M (metastases)	(0) Absence	No metastases	
	(1) Presence	Metastases. Especially on the liver (35%), the lungs (20%), bones (9%), and adrenal glands (5%)	

**Table 2:** Summary of radiological findings in a TNM approaching for gastric cancer.

TNM approaching for gastric cancer		PNCT findings	PET findings
T (primary tumor)	Is (in situ)	Carcinoma in situ. Could be normal at pneumo-PET-CT	
	1	Wall thickness (>3 mm)	Hypercaptation of FDG (it depends on the size of the tumor)
	2		
	3	Blurred serosal contour, strand-like areas of increased attenuation on the perigastric fat	
	4	Obliteration of fat planes with adjacent structures: T4a: visceral peritoneum only T4b: T4b adjacent structures Tumor spread frequently occurs via ligamentous and peritoneal reflections to adjacent organs	

N (lymph node involvement)	(0) Absence	Normal lymph nodes (smooth, well-defined border, uniform homogeneous attenuation, and a central fatty hilum, moderate and homogeneous enhancement, no FDG uptake)	
	(1) One or two nodes	Larger than 10 mm for abdominal nodes, or larger than 5 mm for supraclavicular nodes Morphology alterations, central necrosis, or marked or heterogeneous enhancement should also be considered	Hypercaptation of FDG (even in normal-sized nodes should be considered pathological)
	(2) Three to six nodes		
	(3) Seven or more nodes		

M (metastases)	(0) Absence	No metastases	
	(1) Presence	Metastases	

## Data Availability

The individual patient data used to support the findings of this study have not been made available in order to protect patient privacy.

## Abbreviations

PNCT: Pneumo-computed tomography
PET: Positron emission tomography
FDG: 2-(Fluorine-18)fluoro-2-deoxy-D-glucose
EGJ: Esophagogastric junction
GIST: Gastrointestinal stromal tumors
AJCC: American Joint Committee of Cancer
CE: Conventional endoscopy
BS: Barium studies
EUS: Endoscopic ultrasound.

## Additional Points

*Key Points*. Pneumo-PET-CT is a new technique that combines pneumo-computed tomography with positron emission tomography. Pneumo-PET-CT appears to be a useful hybrid scanning-based technique on the presurgical and after neoadjuvant therapy evaluation of esophageal, esophagogastric junction (EGJ), and gastric tumors in a single shot study. Pneumo-PET-CT allows a simultaneous noninvasive assessment of the esophageal and gastric wall and distant metastatic disease (including the PET-FDG benefits) in a sole imaging method, minimizing both radiation and iodinated-contrast media exposure and reducing costs for the health system.
